# Development of a data-driven approach to Adverse Outcome Pathway network generation: a case study on the EATS-modalities

**DOI:** 10.3389/ftox.2023.1183824

**Published:** 2023-05-09

**Authors:** Linus Wiklund, Sara Caccia, Marek Pípal, Penny Nymark, Anna Beronius

**Affiliations:** ^1^ Institute of Environmental Medicine, Karolinska Institutet, Stockholm, Sweden; ^2^ Università degli Studi di Milano, Milano, Italy

**Keywords:** AOP, AOP network, EATS-modalities, endocrine disruptors, ED assessment, computational, data-driven

## Abstract

Adverse Outcome Pathways (AOPs) summarize mechanistic understanding of toxicological effects and have, for example, been highlighted as a promising tool to integrate data from novel *in vitro* and *in silico* methods into chemical risk assessments. Networks based on AOPs are considered the functional implementation of AOPs, as they are more representative of complex biology. At the same time, there are currently no harmonized approaches to generate AOP networks (AOPNs). Systematic strategies to identify relevant AOPs, and methods to extract and visualize data from the AOP-Wiki, are needed. The aim of this work was to develop a structured search strategy to identify relevant AOPs in the AOP-Wiki, and an automated data-driven workflow to generate AOPNs. The approach was applied on a case study to generate an AOPN focused on the Estrogen, Androgen, Thyroid, and Steroidogenesis (EATS) modalities. A search strategy was developed *a priori* with search terms based on effect parameters in the ECHA/EFSA Guidance Document on Identification of Endocrine Disruptors. Furthermore, manual curation of the data was performed by screening the contents of each pathway in the AOP-Wiki, excluding irrelevant AOPs. Data were downloaded from the Wiki, and a computational workflow was utilized to automatically process, filter, and format the data for visualization. This study presents an approach to structured searches of AOPs in the AOP-Wiki coupled to an automated data-driven workflow for generating AOPNs. In addition, the case study presented here provides a map of the contents of the AOP-Wiki related to the EATS-modalities, and a basis for further research, for example, on integrating mechanistic data from novel methods and exploring mechanism-based approaches to identify endocrine disruptors (EDs). The computational approach is freely available as an R-script, and currently allows for the (re)-generation and filtering of new AOP networks based on data from the AOP-Wiki and a list of relevant AOPs used for filtering.

## 1 Introduction

The Adverse Outcome Pathway (AOP) framework summarizes mechanistic understanding of toxicological effects and is, for instance, considered a promising tool for integrating data from novel *in vitro* and *in silico* methods. These methods may also be referred to as New Approach Methodologies (NAMs), in the regulatory setting. AOPs organize and structure toxicological knowledge and describe how the perturbation of a biological unit may be causally linked to an adverse outcome (Ankley et al., 2010). As such, AOPs consist of a chain of key events (KEs) causally linked together via key event relationships (KERs), until an adverse outcome (AO) is reached. These pathways are chemical agnostic; the molecular initiating event (MIE) can in principle be triggered by a number of stressors. The Organisation of Economic Cooperation and Development (OECD) has developed a Guidance Document (OECD, 2017) and accompanying Handbook (Society for Advancement of AOPs, 2018) to promote harmonized and scientifically robust development and evaluation of AOPs. The AOP-Wiki (https://aopwiki.org/) is a database where AOPs, KEs and KERs at different stages of development are described and shared. The contents of the AOP-Wiki are crowd-sourced, and research groups and organizations around the world are jointly contributing to developing new AOPs and refining existing ones. Since the components of AOPs are modular, and may be part of several AOPs simultaneously, complexity and interconnectedness of the AOP-Wiki contents is constantly evolving.

Mapping the contents of the AOP-Wiki allows for the construction of AOP networks, by identifying MIEs, KEs or AOs that are shared by several AOPs and can provide nodes for connecting AOPs together, resulting in an AOP network (AOPN). When the AOP framework is applied in practice, AOPNs are usually needed to describe the complexity and interconnections of toxicological pathways and effects. These networks are developed on a case-by-case basis for specific problem formulations and can have different scope and structures in order to be fit-for-purpose (Knapen et al., 2018). It is also possible to construct AOPNs in different ways, either by combining already existing AOPs at shared KEs, or by developing a full network from the start that includes several novel AOPs (Knapen et al., 2018). There are currently no harmonized principles or guidance available for the construction of AOPNs, and it is up to the user to apply the most appropriate approach in each case. Lack of a harmonized approaches hamper comparisons between networks for the same or similar toxicological effects, and likely contributes to an inefficient workflow as methodologies have to be developed *de novo* in each case.

With the current number of AOPs in the AOP-Wiki, it is possible to manually screen the AOP-Wiki, identify relevant AOPs, and extract any parameters needed to construct a fit-for-purpose AOP network. However, with the growing number of AOPs, this expert-driven approach will become more time-consuming and complex. Instead, data-driven approaches to standardize and shorten the process, combined with expert-driven approaches to maintain high quality and fit-for-purpose networks, will most likely be needed. Establishing a structured search strategy to identify relevant AOPs can reduce the time spent screening the AOP-Wiki as the number of AOPs increases. A data-driven approach that automatically formats and filters the AOP-Wiki data, and allows for import into a network visualization software, can automate and thus greatly facilitate the data extraction.

As a case study, we chose to examine AOPs related to endocrine disruptors (EDs). Adverse effects on human health and the environment from EDs, is an issue of high concern in the European Union and globally, and several regulatory measures have been taken in the past decade to reduce exposure to such compounds (European Commission, 2018a; European Commission, 2020). Scientific criteria for identifying chemicals with endocrine disrupting properties have been implemented for plant protection products (PPPs) and biocidal products (BPs) in the EU (European Commission, 2017; European Commission, 2018b), based on the WHO definition of an ED (European Commission, 2020) and state that to be identified as an ED, a chemical must fulfill all of the following criteria.

1. show an adverse effect in an intact organism or its progeny;
2. have an endocrine mode of action, i.e., alter the function(s) of the endocrine system;
3. and the observed adverse effect is a consequence of the endocrine mode of action.

AOPs and AOPNs can be useful tools when identifying EDs, especially for Mode of Action (MoA) analysis to establish the causal relationship between an endocrine mechanism and an adverse outcome, criterium number 3 above (Audouze et al., 2021). AOP(N)s may also be used as basis for identification of critical endocrine related KEs that should be targeted for testing and, consequently, development of novel methods that are relevant for ED assessments. Currently, a significant amount of *in vivo* animal data is required to fulfill the scientific ED criteria. At the same time, the EU has expressed the need and intention to reduce and replace animal studies in regulatory toxicity testing (The European Parliament and the Council of the European Union, 2010). There are also concerns regarding the adequacy of current standardized animal studies to identify endocrine-mediated effects relevant to humans and that traditional toxicity endpoints may fail to detect endocrine disruptors (Johansson et al., 2021). Many research and regulatory efforts are ongoing to develop novel methods, or NAMs such as *in vitro* and *in silico* approaches, that provide mechanistic information on endocrine-mediated pathways. However, these novel methods are difficult to integrate into regulatory risk assessments due to, for example, uncertainties surrounding non-validated methods and the relevance of the mechanistic data generated for assessing adverse health outcomes (Browne et al., 2019). Furthermore, animal-free test methods and models cannot replace animal tests on a one-to-one basis, and there is a need to develop and apply batteries of tests, such as defined approaches (DA) and integrated approaches to testing and assessment (IATAs) (Browne et al., 2020). To improve regulatory ED assessment, there is need for new strategic approaches that facilitate inclusion of mechanistic data, also from non-standardized and novel methods, and that are anchored in a robust understanding of endocrine-mediated toxicity pathways. By applying AOPNs in this assessment, mechanistic data from different NAMs with corresponding KEs in the network could be integrated and reliably linked to downstream adverse effects.

The aims of this work were to 1) develop a structured search strategy to identify relevant AOPs in the AOP-Wiki, 2) develop an automated, generally applicable data-driven approach to AOPN generation, based on data from the AOP-Wiki, and 3) to apply the approach in a case study on estrogen, androgen, thyroid and steroidogenesis (EATS)-related endocrine mechanisms and EATS-mediated adversity. Although endocrine disruption through the EATS-modalities was chosen as a case study for this project, the goal was to develop an approach that can be generally applied in different projects by searching for and defining a list of AOPs relevant to a specific problem formulation. The choice of EATS as a case study was driven by the current regulatory focus on these modalities, in the context of identification of EDs in the EU (Andersson et al., 2018).

## 2 Materials and methods

### 2.1 Identification of relevant AOPs from the AOP-Wiki

The approach described here is based on development of an AOPN from already existing AOPs. Also, this approach is generally applicable, and each step must be adapted to the specific problem formulation, exemplified in Table 1. The first step is thus to identify relevant AOPs from the AOP-Wiki. Defining a search strategy and deciding which search terms to include will differ depending on the problem formulation in each case. The final selection of relevant AOPs will require further filtration of AOPs based on, e.g., taxonomic or sex applicability. In the case study, AOPs relevant for EATS-mediated activity or adversity were identified from the AOP-Wiki by conducting full-text searches in AOP-pages. Pre-defined search terms were formulated based on the *in vitro* mechanistic parameters, *in vivo* mechanistic parameters, and EATS-mediated parameters listed in tables 12–17 in the ECHA/EFSA ED Guidance Document (Andersson et al., 2018). The complex syntax of certain parameters in the ED Guidance Document rendered them inappropriate as search terms, and syntax alignment and simplification of these parameters had to be made prior to the search in the AOP-Wiki. The EATS-modalities are well preserved across vertebrate species, and disruption of these modalities observed in non-mammals, especially early KEs, is informative for the conclusion about ED properties also for mammals, and *vice versa*. Hence, the inclusion of EATS-related AOPs was not restricted to those with relevance for human health only and AOPs with an overall applicability for non-mammals (fish and amphibians) were also included. A first search was performed on 2021–04-09 using the established search terms. A second search was conducted on 2023–02-03 to include any new AOPs added since the first search. The full list of search terms used, and the parameters of the ED Guidance Document they were based on, can be found in the Supplementary Materials.

**TABLE 1:** The steps of the general approach and the corresponding method used for the EATS case study.

General approach	EATS case study
Define criteria for AOP selection	Pre-defined search terms were developed based on parameters in the ED Guidance Document. No further filters regarding applicability domain or developmental stage were utilized
Query AOP-Wiki and extract AOPs	Data was downloaded from the AOP-Wiki. A search was performed to identify initially relevant AOPs.
Refine AOP selection	Information on each AOP was screened based on its information in the Wiki, and a final list of relevant AOPs was generated.
Download and process data automatically	Data was downloaded from the AOP-Wiki and processed in R.
Generate AOP network	The AOPN was generated in Cytoscape
Identify issues and refine network	Missing KERs were identified and resolved manually by investigating the AOPs in the AOP-Wiki.

AOPs retrieved from the AOP-Wiki by the search strategy were subsequently screened to establish a list of relevant AOPs. The Abstract, Background and Overall Assessment section of each AOP were carefully reviewed, and the graphical representation of each AOP was analyzed. If the AOP was deemed irrelevant for any of the EATS-modalities, it was excluded. The relevance of each AOP was investigated by affirming that KEs based on the established search terms were present in the AOP, and not only mentioned in the abstract or background of the Wiki page. For example, the initial search retrieved some AOPs where a predefined search term was only present in the abstract or background but was overall irrelevant for the mechanisms or effects described by the AOP itself. In such cases, the AOP was not included. A low level of development of the AOP was not used as a criterion for exclusion at this stage. Uncertainties regarding the inclusion or exclusion of specific AOPs were resolved by discussion in the project group. In principle, the process was to include any AOP that may be also indirectly relevant, in order to avoid accidentally excluding potentially relevant AOPs. This is important since the purpose of the network was to show the overall contents of the AOP-Wiki related to EATS, and then further filter the network depending on the purpose of future research questions. Following this screening step, a list of AOPs considered relevant for the EATS-modalities was constructed.

### 2.2 Data processing in R

Three tab-separated values (TSV) formatted files with a snapshot of the AOP-Wiki contents were downloaded from the AOP-Wiki on 2021–07-04, and a first version of the network was created. Another search was performed on 2023–02-03 to update the network, and new data was downloaded from the AOP-Wiki on 2023–02-10. The latest version of the data (from 2023 to 02–10) was used to create the final network presented in the paper. The data downloaded from the Wiki after both searches are available in the Supplementary Material. When downloading files from the AOP-Wiki, the data is divided into three separate files each containing a subset of the wiki contents. They were imported into R-studio (https://www.rstudio.com/) to be combined and formatted for import into Cytoscape. The Key Events file contain information on the KEs (AOP ID, KE ID, Event type, and Event name), the Key Event Relationship file contain information on the KERs (AOP ID, upstream event ID, downstream event ID, relationship ID, adjacency, relationship evidence, and quantitative understanding of the relationship) and the Key Event Component file contains detailed information on the object of the KE (for example, a receptor or signaling molecule) and its related process (AOP ID, KE ID, direction of effect, object source, object ontology ID, object term, process source, process ontology ID, and process term). To construct networks specifically for EATS- mediated effects, the data from the AOP-Wiki was filtered based on the list of identified relevant AOPs described in section 2.1. After the processing and filtering was done, the resulting joined table was exported as a semicolon separated file. The script file with the R code and comments is available for download at GitHub (https://github.com/linuwi-ki/AOPN-Generation).

Briefly, the files were formatted to allow for appropriate processing, for example, by removing text from columns with IDs (e.g., from “AOP:42” to “42” in the AOP ID column). The evidence and quantitative understanding of KERs were changed to text-based categories instead of numerical ones. The data was then filtered to exclude any AOP IDs that were not present in the list of relevant AOPs generated in section 2.1. The values for object and process source, as well as ontology ID and ontology term in the KEC file were merged and duplicates were removed. The three separate tables were then merged into one joined table and any remaining duplicates were removed before the final table was exported as an CSV file. This file was imported into Cytoscape to visualize the network.

### 2.3 AOP network generation

After processing of the data in R and filtration to only include EATS-related AOPs, the generated CSV file was imported into Cytoscape for visualization. A critical part of this process is to correctly establish the nodes, represented by KEs, and edges, represented by KERs, in the software. This was done by identifying a Source node (upstream KE ID) and a Target node (downstream KE ID) in each row of the table. As a result, Cytoscape visualized all the KEs and the relationships between the upstream KEs and downstream KEs of all AOPs. Since KEs in AOPs are modular and can be connected to several downstream or upstream KEs, a network of connected events is constructed. The initial arrangement of the network was performed by applying the yFiles Layout Algorithm for Cytoscape (https://www.yworks.com/products/yfiles-layout-algorithms-for-cytoscape) using the yFiles Hierarchic Layout preset. Manual modifications were then performed to the layout, including changes to the color palette, as well as deciding what information to display in the nodes and edges. It should be noted that all parameters extracted from the AOP-Wiki were not visualized in the figure but may be integrated if needed depending on the research question. For example, gene ontology information, and the level of evidence and quantitative understanding of KERs, can be visualized in the nodes and edges, respectively.

During the network generation, missing KERs were identified in certain AOPs. A KER will be missing in the data-driven network in Cytoscape if it has not been properly described and entered into the AOP-Wiki. For example, if it is only mentioned in the “Summary of the AOP” or depicted in the graphical representation. One reason might be that the AOP is in early stages of development. Thus, whenever a missing link between KEs was identified, the contents of the AOP page in the AOP-Wiki (Abstract, Background, or Overall Assessment section, or the graphical representation of the AOP) were screened for relevant information to identify missing KERs. If identified, missing KERs were manually added to the network and visualized in Cytoscape but do not have a Relationship ID since they did not exist in the AOP-Wiki when the data were downloaded.

## 3 Results

### 3.1 The overall approach

**FIGURE 1:**
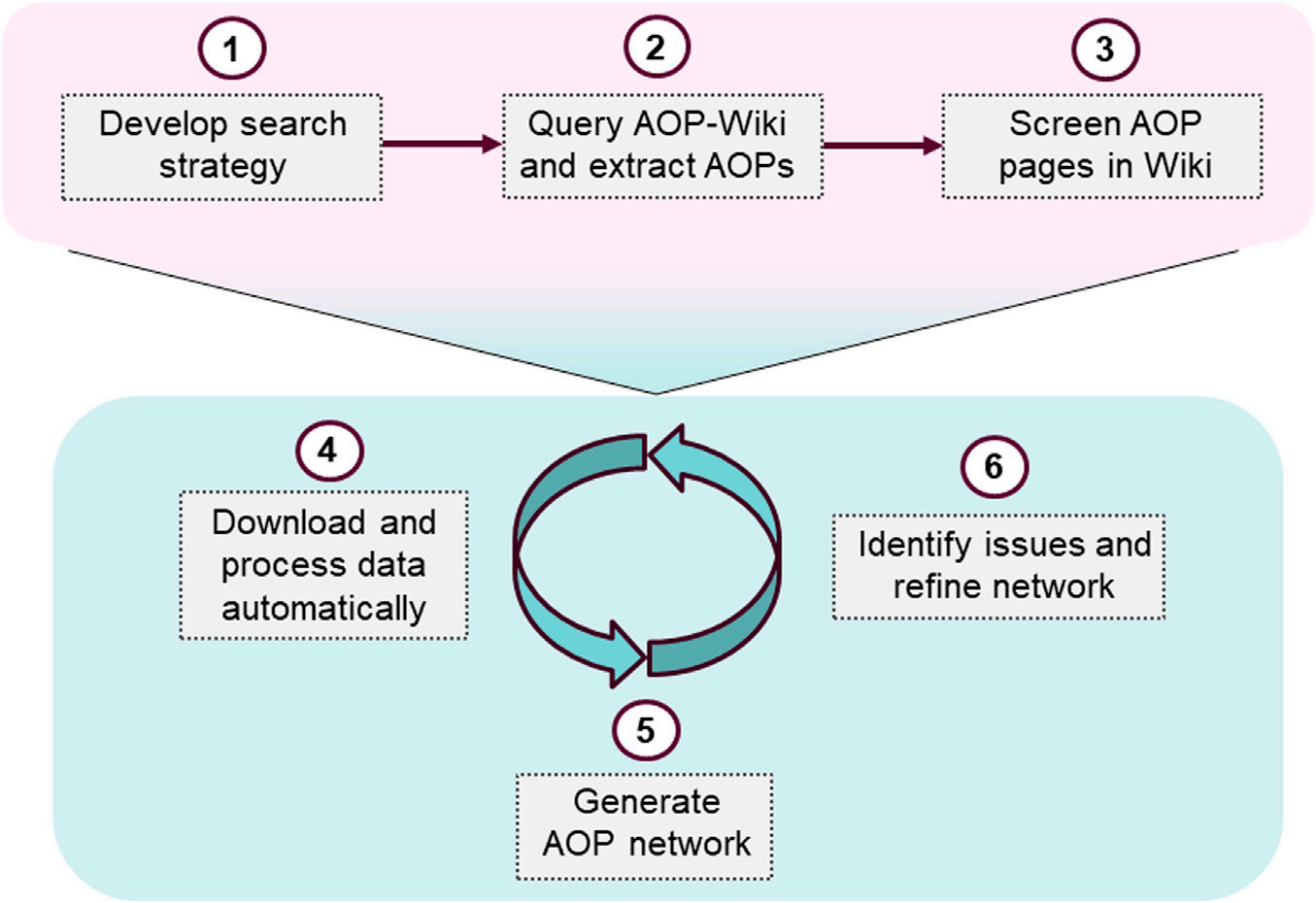
Schematic representation of the overall approach to generate a curated AOPN.

A generally applicable approach to generate AOP networks was developed and applied in a case study on the EATS-modalities. A structured search strategy to identify AOPs in the AOP-Wiki was implemented by identifying relevant search terms in the ED Guidance document (Andersson et al., 2018). A data-driven workflow to process and format data for visualization in Cytoscape was produced, resulting in an R-script. The script allows for automatic processing of the data from the AOP-Wiki and requires very little prior experience in R and R-studio to apply. Though the focus of this paper is on endocrine disruption, the approach presented is generally applicable and can be used to investigate other adverse effects, by supplying a list of AOPs relevant to the problem formulation, to be included in the network. The network can also be updated by identifying new relevant AOPs and downloading new data from the AOP-Wiki. The process of identifying relevant AOPs in the AOP-Wiki, and subsequent network generation and refinement, is summarized in Figure 1.

### 3.2 Identification of relevant AOPs from the AOP-Wiki

**FIGURE 2:**
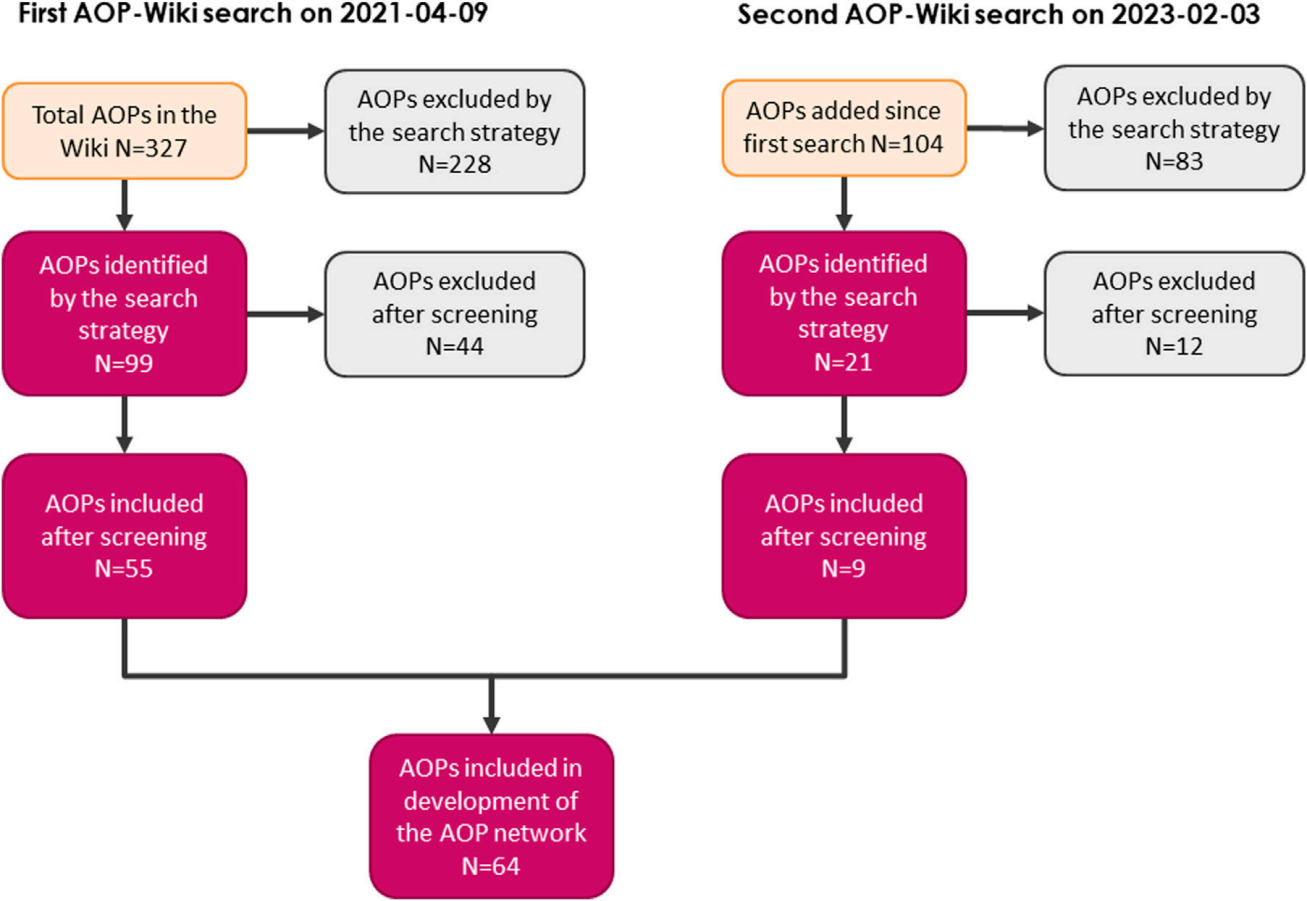
Flowchart depicting the identification and screening of EATS-relevant AOPs, and the number of AOPs in each step of the process.

Following the first search of the AOP-Wiki using predefined search terms, 99 AOPs were retrieved as potentially relevant for the EATS-modalities. After curating the data and careful review of the wiki entries, 44 AOPs were excluded and a total of 55 AOPs were included as relevant for the construction of an EATS-related AOPN. After the second search, a total of 64 AOPs were included in the generation of the AOPN (Table 2). Most AOPs shared at least 1 KE, and a large network was formed; the main network. However, 16 AOPs (non-shaded rows in Table 2) were not connected to the main network and either may have been connected with one or a few AOPs to create a smaller sub-network, or not connected to any other AOPs at all. Some AOPs also existed only as separate KEs, since KERs had not been added properly in the wiki. Early-stage AOPs may also be empty in the AOP-Wiki, and therefore no data was retrieved from these AOPs although they were considered relevant during the filtration step. Reasons for exclusion of AOPs during the screening process were commonly that one or several of the search terms appeared in the descriptions of AOPs that were not endocrine related. A flowchart of the number of included and excluded AOPs in each step is described in Figure 2. A more detailed version of Table 2 containing the IDs of AOPs identified as relevant, their life-stage, taxonomic and sex applicability, and OECD status are listed in Supplementary Material, and provide a summary of EATS-related AOPs in the AOP-Wiki available at the time of the second search (2023–02–03).

**TABLE 2:** Summary of the 64 selected AOPs extracted from the AOP-wiki. AOPs in shaded rows are connected to the main network.

AOP ID	Title^1^
**7**	*Aromatase (Cyp19a1) reduction leading to impaired fertility in adult female*
**8**	*Upregulation of Thyroid Hormone Catabolism via Activation of Hepatic Nuclear Receptors, and Subsequent Adverse Neurodevelopmental Outcomes in Mammals*
**18**	*PPARα activation in utero leading to impaired fertility in males*
**19**	*Androgen receptor antagonism leading to adverse effects in the male foetus (mammals)*
**23**	*Androgen receptor agonism leading to reproductive dysfunction (in repeat-spawning fish)*
**25**	*Aromatase inhibition leading to reproductive dysfunction*
**29**	*Estrogen receptor agonism leading to reproductive dysfunction*
**30**	*Estrogen receptor antagonism leading to reproductive dysfunction*
**42**	*Inhibition of Thyroperoxidase and Subsequent Adverse Neurodevelopmental Outcomes in Mammals*
**54**	*Inhibition of Na+/I- symporter (NIS) leads to learning and memory impairment*
**110**	*Inhibition of iodide pump activity leading to follicular cell adenomas and carcinomas (in rat and mouse)*
**111**	*Decrease in androgen receptor activity leading to Leydig cell tumors (in rat)*
**117**	*Androgen receptor activation leading to hepatocellular adenomas and carcinomas (in mouse and rat)*
**119**	*Inhibition of thyroid peroxidase leading to follicular cell adenomas and carcinomas (in rat and mouse)*
**124**	*HMG-CoA reductase inhibition leading to decreased fertility*
**128**	*Kidney dysfunction by decreased thyroid hormone*
**134**	*Sodium Iodide Symporter (NIS) Inhibition and Subsequent Adverse Neurodevelopmental Outcomes in Mammals*
**146**	*Interference with thyroid serum binding protein transthyretin and subsequent adverse human neurodevelopmental toxicity*
**152**	*Interference with thyroid serum binding protein transthyretin and subsequent adverse human neurodevelopmental toxicity*
**153**	*Aromatase Inhibition leading to Ovulation Inhibition and Decreased Fertility in Female Rats*
**155**	*Deiodinase 2 inhibition leading to increased mortality via reduced posterior swim bladder inflation*
**156**	*Deiodinase 2 inhibition leading to increased mortality via reduced anterior swim bladder inflation*
**157**	*Deiodinase 1 inhibition leading to increased mortality via reduced posterior swim bladder inflation*
**158**	*Deiodinase 1 inhibition leading to increased mortality via reduced anterior swim bladder inflation*
**159**	*Thyroperoxidase inhibition leading to increased mortality via reduced anterior swim bladder inflation*
**162**	*Enhanced hepatic clearance of thyroid hormones leading to thyroid follicular cell adenomas and carcinomas in the rat and mouse*
**165**	*Antiestrogen activity leading to ovarian adenomas and granular cell tumors in the mouse*
**167**	*Early-life estrogen receptor activity leading to endometrial carcinoma in the mouse.*
**175**	*Thyroperoxidase inhibition leading to altered amphibian metamorphosis*
**176**	*Sodium Iodide Symporter (NIS) Inhibition leading to altered amphibian metamorphosis*
**188**	*Iodotyrosine deiodinase (IYD) inhibition leading to altered amphibian metamorphosis*
**189**	*Type I iodothyronine deiodinase (DIO1) inhibition leading to altered amphibian metamorphosis*
**190**	*Type II iodothyronine deiodinase (DIO2) inhibition leading to altered amphibian metamorphosis*
**191**	*Type III iodotyrosine deiodinase (DIO3) inhibition leading to altered amphibian metamorphosis*
**200**	*Estrogen receptor activation leading to breast cancer*
**271**	*Inhibition of thyroid peroxidase leading to impaired fertility in fish*
**288**	*Inhibition of 17α-hydrolase/C 10,20-lyase (Cyp17A1) activity leads to birth reproductive defects (cryptorchidism) in male (mammals)*
**289**	*Inhibition of 5α-reductase leading to impaired fecundity in female fish*
**295**	*Early-life stromal estrogen receptor activation by endocrine disrupting chemicals in the mammary gland leading to enhanced cancer risk*
**300**	*Thyroid Receptor Antagonism and Subsequent Adverse Neurodevelopmental Outcomes in Mammals*
**305**	*5α-reductase inhibition leading to short anogenital distance (AGD) in male (mammalian) offspring*
**306**	*Androgen receptor (AR) antagonism leading to short anogenital distance (AGD) in male (mammalian) offspring*
**307**	*Decreased testosterone synthesis leading to short anogenital distance (AGD) in male (mammalian) offspring*
**309**	*Luteinizing hormone receptor antagonism leading to reproductive dysfunction*
**314**	*Binding to estrogen receptor (ER)-α in immune cells leading to exacerbation of systemic lupus erythematosus (SLE)*
**321**	*Reduced environmental pH leading to thinner shells in Mytilus edulis*
**344**	*Androgen receptor (AR) antagonism leading to nipple retention (NR) in male (mammalian) offspring*
**345**	*Androgen receptor (AR) antagonism leading to decreased fertility in females*
**346**	*Aromatase inhibition leads to male-biased sex ratio via impacts on gonad differentiation*
**348**	*Inhibition of 11β-Hydroxysteroid Dehydrogenase leading to decreased population trajectory*
**349**	*Inhibition of 11β-hydroxylase leading to ecreased population trajectory*
**366**	*Competitive binding to thyroid hormone carrier protein transthyretin (TTR) leading to altered amphibian metamorphosis*
**367**	*Competitive binding to thyroid hormone carrier protein thyroid binding globulin (TBG) leading to altered amphibian metamorphosis*
**372**	*Androgen receptor antagonism leading to testicular cancer*
**376**	*Androgen receptor agonism leading to male-biased sex ratio*
**393**	*AOP for thyroid disorder caused by triphenyl phosphate*
**401**	*G protein-coupled estrogen receptor 1 (GPER) signal pathway in the endocrine disrupting effect*
**402**	*Thyroid peroxidase (TPO) inhibition leads to periventricular heterotopia formation in the developing rat brain*
**440**	*Hypothalamus estrogen receptors activity suppression leading to ovarian cancer via ovarian epithelial cell hyperplasia*
**443**	*Alcohol Induced DNA damage and mutations leading to Metastatic Breast Cancer*
**445**	*Estrogen Receptor Alpha Agonism leads to Impaired Reproduction*
**465**	*Alcohol dehydrogenase leading to reproductive dysfunction*
**476**	*Adverse Outcome Pathways diagram related to PBDEs associated male reproductive toxicity*
**477**	*Androgen receptor (AR) antagonism leading to hypospadias in male offspring*

^1^The information for AOP IDs, 7–376 was collected on 2022–09-13 and AOP, titles may have been modified since the initial search for data in the AOP-Wiki on 2021–07-04. AOP IDs, higher than 376 were collected on 2023–02-21, but may also have been changed since.

### 3.3 AOP network generation

**FIGURE 3:**
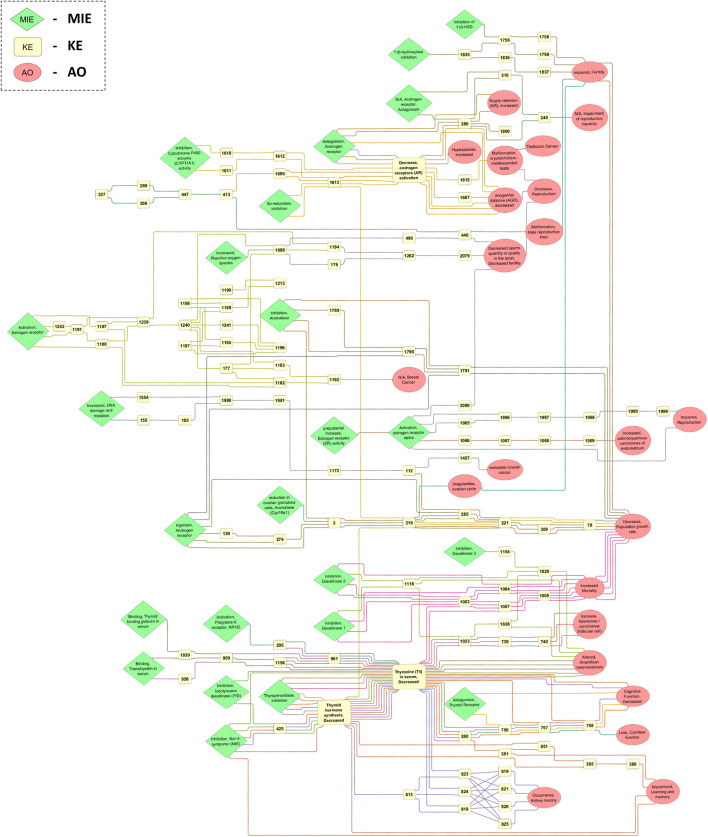
Main EATS-network containing KEs from 48 AOPs, including 24 MIEs, 114 kEs and 23 AOs. The direction of the AOPs is from left to right, and each KER is colored based on the AOP ID it belongs to. The label of MIEs, AOs and certain KEs represent the name of the event in the AOP-Wiki, while most KEs only contain the KE ID.

The EATS-related AOP network was generated in Cytoscape based on the extracted and processed data from the AOP-Wiki. The network presents AOPs in a horizontal way, with MIEs and early KEs to the left, and late KEs and AOs to the right. Only the main network was visualized to simplify the figure, but high resolution images of the main network, and of any sub-networks, single AOPs, and single KEs, can be found in the Supplementary Material. During the network generation, it was also discovered that some KEs seem to describe the same biological event, but with slight differences in terminology of the KE title. These duplicate KEs may exist in the database as separate entries due to having different applicability domains, but could also exist because of differences in terminology. The main AOPN is presented in Figure 3, where MIEs, AOs and certain highly connected KEs have been highlighted.

### 3.4 Manual additions

KERs were found to be missing in the generated AOP network for 8 AOPs. The included AOPs that were modified were: 200, 372, and 476. The KERs were only added into Cytoscape to allow for proper network generation. However, since no information on the evidence level could be retrieved from the graphical abstract or background or the AOP page, these KERs have no information on level of evidence or quantitative understanding. The specific KERs that were added, and a detailed explanation of the changes, can be seen in Table 3. Clear KERs could not be identified for 5 of the 8 AOPs (152, 153, 393, 401, 402) in any section of the AOP-Wiki page for each AOP, and therefore no manual additions were made to these AOPs. A figure of all single AOPs and KEs can be found in the Supplementary Material.

**TABLE 3:** Summary of the manual additions made to the AOP network in Cytoscape based on information present in the free text or graphical abstract on each AOP page in the AOPWiki, but that was not included in the downloaded data.

AOP IDs	KE IDs	Addition of KERs
200	177, 1195, 1197, 1198	• Added a relationship from ID 1240 to 177, and from 177 to 1088, 1182, and 1183.
		• From 1197 to 1198 to 1195.
		• From 1195 to 1196.
		• From 1197 to 1195, 1196, and 1189.
		• From 1198 to 1195, 1196, and 1189.
372	1839	• Added a relationship from ID 1616 to 1839.
476	176, 496, 520, 1115, 1262, 1863, 2079, 2080	• Added a relationship from ID 1088 to 176, and from 176 to 1262.
		• From 1088 to 496, and from 496 to 446.
		• From 446, 2079, and 2080 to 520, and from 520 to 1863.
		• From 1115 to 1088.
		• From 176 to 1194 to 1262, and from 1262 to 2079.
		• From 520, 1616, and 1688 to 1863.
		• From 1262 to 2079, and from 2079 to 520.
		• From 1065 to 2080, and from 2080 to 520.

## 4 Discussion

In this work, a general approach for automated development of AOPNs is proposed and illustrated using a case study on an AOPN for the EATS-modalities of endocrine disruption. AOP networks are often needed for practical applications of the AOP framework (Villeneuve et al., 2014) and different networks are required for different purposes. The data-driven method described herein was developed with the intent of making it easy to construct new networks for different purposes, and to be able to update networks with new data as the AOP-Wiki grows. Adding layers of complexity or filters to focus on specific aspects is not covered by this paper, but is needed to make the network fit for purpose for specific applications (Knapen et al., 2018). Apart from data layering not being within the scope of this study, AOP-Wiki contents complying with the Findability, Accessibility, Interoperability, and Reuse (FAIR) principles (Wilkinson et al., 2016) is important. Making sure that the AOP-Wiki data are aligned with the FAIR principles, and especially the I—interoperability, is crucial for data layering as the AOP-Wiki grows. Indeed, the importance of the FAIR principles, and interoperability specifically, has been discussed previously. For example, in the context of utilization of ‘omics data for AOP development and increased regulatory use of AOPs (Martens et al., 2018). Also, in the structuring of the AOP-Wiki database to ease the use of computational measures in exploring and applying AOPs (Angeler et al., 2019; Martens et al., 2022). In line with the conclusions of these authors, the work presented herein shows that redundancy of KEs is still an issue, and AOP-Wiki machine-readability still require improvements to allow for computational approaches to be applied.

A network can be generated in the beginning of a project to support, for example, the development of a search strategy based on central pathways or KEs in the network specifically relevant for the research question. Then, the network may be updated and refined whenever needed during the course of the study, to analyze or discuss results with the most recent landscape of AOPs from the AOP-Wiki. A generally applicable, data-driven and harmonized approach to AOPN generation allows for comparisons between networks of the same or similar effects and streamlines the updating of networks when new data is available. Also, it might facilitate the application of AOP(N)s where the methodology would otherwise have to be developed *de novo* for each project. This data-driven approach standardizes and shortens the network generation process, while the expert-driven data collection and network refining maintains a high quality and fit-for-purpose network.

As new AOPs are developed, and the AOP-Wiki grows, it will become more desirable to set up structured search strategies to retrieve the AOPs relevant for construction of AOP networks. In the approach to AOP network development described here, a first step was to identify potentially relevant AOPs from the AOP-Wiki using predefined search terms. The AOP-Wiki search strategy developed for this case study can be used as an example and guide for future projects. The search strategy was designed to be broad and inclusive, to provide a sensitive search and not risk missing any potentially relevant AOPs. Consequently, in the subsequent steps almost half of the AOPs retrieved in the search were identified as not relevant and excluded. An alternative approach would have been to manually screen each AOP included in the AOP-Wiki from the start and select those relevant for inclusion based on specific criteria. Such an approach ensures that all potentially relevant AOPs are included for the construction of the network, provided that clear inclusion/exclusion criteria are established beforehand. As the number of AOPs in the Wiki is still limited (327 at the time of the first search for this project, and 431 at the time of the second search) screening all AOPs is certainly a manageable approach as of right now.

The resulting list of relevant AOPs from the search strategy was used to filter data downloaded from the AOP-Wiki, to specify the scope of the network. Data from the Wiki were first processed, and then filtered, automatically using a script, which is freely available and generalizable to any type of AOP network by defining the list of AOPs to be included. This data-driven approach standardizes and speeds up the process of generating AOPN and allows for re-generation of networks when AOPs in the Wiki are added or updated, while maintaining the specific purpose of the network. The table produced by the script can be imported into Cytoscape to visualize the AOPN. This initial, data-driven, visualization of the AOPN can subsequently be refined as needed for the specific use, for example, by data layering as described previously.

For an AOP network to be applicable for its specific purpose, it often needs to be modified. For example, Knapen et al. have shown how filters and layers can be implemented to refine AOPN and provide more application-specific networks (Knapen et al., 2018). The network is a basis for future research and must be filtered for specific applications, such as thyroid disruption and brain development. Nonetheless, the AOPN presented in this paper still provide an inventory and visualization of all available EATS-related AOPs in the AOP-Wiki. Although this method is quick and easy to apply, it is noteworthy that any discrepancies present in the AOP-Wiki database will be included in the downloaded data. For example, missing KERs or duplicate KEs which are biologically the same but exist as different entities in the AOP-Wiki due to different naming conventions. These issues must be manually identified and resolved after visualization of the network.

In a solely expert-driven approach, the AOP-Wiki is manually screened for relevant AOPs and the extraction and processing of data is not automated, compared to a data-driven approach where extraction and/or processing of data is automated. Expert-driven approaches allows a more fit-for-purpose data extraction procedure, where specific parameters may be included or excluded depending on what is needed for the project. Also, issues like missing KERs or duplicate KEs may be identified and resolved during the screening process, though it is more time-consuming and may introduce bias and/or additional mistakes in the data. For example, the applicability domain of duplicate KEs may differ, and therefore expert knowledge is required in the decision of merging KEs or not. Therefore, combining the expert-driven approaches to identify relevant AOPs in the AOP-Wiki and to resolve issues and harmonize the data after network visualization, with the data-driven approach to process and format the data, could be the way forward. In this work, the data-driven approach was used to collect and process data from the AOP-Wiki, and an expert approach was used to identify relevant AOPs in the AOP-Wiki by a structured search strategy and manual screening of AOP page contents. A need to allow for searching specific sections of AOP pages was identified, as almost half of the hits retrieved from the full-text search were excluded in the screening process.

AOP networks for different toxicological effects and applications have been previously constructed, e.g., for thyroid hormone disruption (Noyes et al., 2019), tiered testing strategies for thyroid hormone disruption in zebrafish (Knapen et al., 2020), and neurotoxicity (Spinu et al., 2019). Recently, an AOP network relevant to endocrine-mediated perturbations in general was also developed (Ravichandran et al., 2022). In that study, Ravichandran et al. constructed a network on all relevant endocrine-mediated effects, including non-EATS effects, and focused on graph-theoretic analysis of the network. They also performed a rigorous filtration and manual curation of the extracted AOPs to only include high-confidence AOPs, by a set of predefined criteria. In contrast, the case study presented here included all AOPs in the wiki identified by the search strategy as relevant for the description of EATS-mediated pathways, regardless of development status, and gives a broader overview of the available AOPs for these pathways. This inclusive approach was chosen to provide a basis for different applications, for example, to identify knowledge gaps and needs and prioritization for future AOP-development, to identify shared KEs of interest for further study or refinement, or to explore similarities in toxicological pathways across taxa. Shared KEs are events where several pathways converge at and/or diverge from, and are important as potential biomarkers for effect or targeted for the development of NAMs and IATAs (Knapen et al., 2020). Depending on the intended use, the network can later be refined or simplified on a case-by-case basis in order to be fit for purpose. For example, the EATS-network presented here could be further filtered to specifically focus on a specific endocrine modality. There is currently work ongoing on the use of a thyroid-specific AOPN for identification of ED properties of perfluorooctanesulfonic acid (PFOS). The network is used to develop a search strategy for PFOS based on thyroid disruption and brain development, and the evidence retrieved is mapped back onto the AOPN to identify possible ED MoAs based on empirical data after PFOS exposure. The network may also be useful in identifying data gaps and missing KEs in the AOP-Wiki data.

Developing AOPs is very time-consuming and require large amounts of data to be generated, at different levels of biological organization. To alleviate this, it has been suggested that KERs, instead of full AOPs, can be developed and then framed within a full AOP to allow for regulatory use (Svingen et al., 2021). There are also tools like the AOP-helpFinder (Jornod et al., 2022), which allows for identification of relationships between stressors and biological events from the open literature based on abstracts in PubMed. Tools like these may expedite the growth in the number of AOPs in the AOP-Wiki, further establishing the need for harmonized and data-driven approaches in the application of AOPs.

Future updates of the AOP-Wiki may lead to changes in the structure of the database, but the structured workflow presented here remains relevant. In this work, we identified some limitations of the AOP-Wiki that specifically hampered the construction of AOP networks. Guidelines are in place to promote harmonization of AOP development and terminology such as the Developer’s Handbook (https://aopwiki.org/handbooks/), however some variability and discrepancies between AOP-, KE- and KER-identification and descriptions become apparent when attempting to apply information from the AOP-Wiki in different cases. For example, during the extraction of relevant AOPs for this work, several KERs were found to be missing in the database and had to be manually corrected in the subsequent AOP network generation. Moreover, duplicate KEs which are biologically identical but with differing terminology, were identified. That is, the same KE may have been added as multiple entries in the AOP-Wiki due to having different naming conventions. For example, KE ID 26 “Antagonism, Androgen receptor” and KE ID 27 “N/A, Androgen receptor, Antagonism”. Although these KEs are biologically the same, they will exist as separate KEs in the network with their own KERs. For the purpose of identifying new AOPs, shared KEs, or possible IATAs, these database issues may have a significant impact on the results. However, duplicate KEs could potentially be merged into a single KE after visualization, adding the different KERs of both events together to create a more connected network. This must be done on a case-by-case basis, since the duplicate KEs may have different applicability domains and might be better separated, depending on the problem formulation. Also, combining KEs based on biological similarity may require expertise in specific fields depending on the scope of the AOP network. In this work, it was decided not to merge duplicate/similar KEs, but this may be relevant when filtering the main network into more specific fit-for-purpose AOP networks. For this purpose, the KE component information containing ontology annotations may be used as a basis for identifying duplicate/similar KEs. Ontologies, such as the gene ontology, are standardized and machine-readable statements on the function of genes across different species. The concept of ontologies for AOPs have been discussed and investigated previously (Whaley et al., 2020; Martens et al., 2022), and this work further supports the need for its continued development. Another AOP-Wiki feature that would simplify the process of updating AOP networks is the possibility to sort or filter AOPs based on the date that the AOP was uploaded or last updated. This would make the identification of new/updated AOPs easier, and the need to perform a search and screening of the Wiki may not be needed unless it is suspected that a large amount of AOPs have been added or updated.

To summarize, in this paper a generally applicable approach to generate AOP networks was presented and applied in a case study on the EATS-modalities. This paper also provides insight to AOP and AOP-Wiki developers regarding the importance of the FAIR principles, and issues like missing KERs and duplicate KEs in support of AOPN development. The methodology and network are intended to be used for further research promoting integration of data from novel *in vitro* and *in silico* methods into regulatory ED assessment, and for development of IATAs for EDs. With the expanding regulatory implementation of NAMs, the need for the AOP framework and development of standardized strategies to integrate data from novel methods also increase. This paper highlights the need for development of harmonized approaches to generate AOPNs, and via a case study on endocrine disruption provide examples of prospective research projects and improvements required to fully utilize AOPNs in the future.

## Data Availability

The original contributions presented in the study are included in the article/Supplementary Material, further inquiries can be directed to the corresponding author.

## Supplementary material

Supplementary Table S1 Search terms.

Supplementary Table S2 Extended summary of AOPs.

Supplementary Data Sheet S1 AOP-Wiki data.

Supplementary Figure S1 High-resolution main network.

Supplementary Figure S2 Single AOPs and KEs.
