# Supplementary material for: Development of a data-driven approach to Adverse Outcome Pathway network generation: a case study on the EATS-modalities
Source: Front Toxicol. 2023 May 9;5:1183824. doi: 10.3389/ftox.2023.1183824 (PMC10203404; doi:10.3389/ftox.2023.1183824)
Supplement: Supplementary file 2 [file Table2.DOCX]

Supplementary Material

Supplementary Table 1. A summary of the AOPs relevant to the EATS-modalitites that was included after the screening process.

| AOP ID^[[1]](#footnote-1)^ | Title^^[[2]](#endnote-1)^^ | Life-stage Applicability^1^ | Taxonomic Applicability^1^ | Sex Applicability^1^ | OECD Status^1^ |
| --- | --- | --- | --- | --- | --- |
| 7 | *Aromatase (Cyp19a1) reduction leading to impaired fertility in adult female* | Adult, reproductively mature (High) | *Rattus norvegicus* (High), *Mus musculus* (Low), *Homo sapiens* (Low) | Female (High) | EAGMST Under Review |
| 8 | *Upregulation of Thyroid Hormone Catabolism via Activation of Hepatic Nuclear Receptors, and Subsequent Adverse Neurodevelopmental Outcomes in Mammals* | Fetal to Parturition (moderate) Nursing Child (moderate) | *Rattus norvegicus* | N.A. | Under Development |
| 18 | *PPARα activation in utero leading to impaired fertility in males* | Development (High) | *Rattus norvegicus* (Moderate), *Mus musculus* (Moderate), *Homo sapiens* (Low) | Male (High) | EAGMST Under Review |
| 19 | *Androgen receptor antagonism leading to adverse effects in the male foetus (mammals)* | Foetal | N.A. | Male | N.A. |
| 23 | *Androgen receptor agonism leading to reproductive dysfunction (in repeat-spawning fish)* | Adult, reproductively mature | *Pimephales promelas* | Female | WPHA/WNT Endorsed |
| 25 | *Aromatase inhibition leading to reproductive dysfunction* | Adult, reproductively mature | *Oryzias latipes* (Moderate), *Danio rerio* (Moderate), *Pimephales promelas* (High) | Female (High) | WPHA/WNT Endorsed |
| 29 | *Estrogen receptor agonism leading to reproductive dysfunction* | Juvenile (High), Embryo (High) | *Pimephales promelas* (High), *Coturnix japonica* (High), *Rana pipiens* (High), *Oryzias latipes* (High), *Danio rerio* (High) | Male (High) | N.A. |
| 30 | *Estrogen receptor antagonism leading to reproductive dysfunction* | Adult, reproductively mature (High) | *Danio rerio, Pimephales promelas, Oryzias latipes* | Female (High) | EAGMST Under Review |
| 42 | *Inhibition of Thyroperoxidase and Subsequent Adverse Neurodevelopmental Outcomes in Mammals* | During brain development (high), development (high) | *Homo sapiens* (high), *Rattus norvegicus* (high), *Mus musculus* (moderate) | Male (high), Female (high) | WPHA/WNT Endorsed |
| 54 | *Inhibition of Na+/I- symporter (NIS) leads to learning and memory impairment* | Foetal (High), Perinatal (High), During brain development (High) | *Homo sapiens* (High), *Rattus sp.* (High) | Male (High), Female (High) | WPHA/WNT Endorsed |
| 110 | *Inhibition of iodide pump activity leading to follicular cell adenomas and carcinomas (in rat and mouse)* | N.A. | *Rattus norvegicus, Mus musculus* | Male | N.A. |
| 111 | *Decrease in androgen receptor activity leading to Leydig cell tumors (in rat)* | N.A. | *Rattus norvegicus* | Male | N.A. |
| 117 | *Androgen receptor activation leading to hepatocellular adenomas and carcinomas (in mouse and rat)* | N.A. | *Mus musculus, Rattus norvegicus* | Male, Female | Under Development |
| 119 | *Inhibition of thyroid peroxidase leading to follicular cell adenomas and carcinomas (in rat and mouse)* | N.A. | *Rattus norvegicus, Mus musculus* | Male, Female | N.A. |
| 124 | *HMG-CoA reductase inhibition leading to decreased fertility* | Fetal (Low) | *Rattus rattus* | Male | N.A. |
| 128 | *Kidney dysfunction by decreased thyroid hormone* | 1 to < 3 months (High), Adults (High) | Sprague Dawley (High), *Homo sapiens* | Male (High), Unspecific (High) | Under Development |
| 134 | *Sodium Iodide Symporter (NIS) Inhibition and Subsequent Adverse Neurodevelopmental Outcomes in Mammals* | Perinatal (high) | *Homo sapiens* (high), *Rattus norvegicus* (high) | Male (high), Female (high) | N.A. |
| 146 | *Interference with thyroid serum binding protein transthyretin and subsequent adverse human neurodevelopmental toxicity* | Development (moderate) | *Rattus norvegicus* (moderate) | Mixed (high) | Under Development |
| 152 | *Interference with thyroid serum binding protein transthyretin and subsequent adverse human neurodevelopmental toxicity* | Development (Moderate) | *Rattus norvegicus* (High) | Mixed (Moderate) | Under Development |
| 153 | *Aromatase Inhibition leading to Ovulation Inhibition and Decreased Fertility in Female Rats* | N.A. | N.A. | N.A. | N.A. |
| 155 | *Deiodinase 2 inhibition leading to increased mortality via reduced posterior swim bladder inflation* | Embryo (High) | *Pimephales promales* (High), *Danio rerio* (High) | Unspecific (Moderate) | EAGMST Approved |
| 156 | *Deiodinase 2 inhibition leading to increased mortality via reduced anterior swim bladder inflation* | Larvae (High) | *Pimephales promales* (High), *Danio rerio* (High) | Unspecific (Moderate) | EAGMST Approved |
| 157 | *Deiodinase 1 inhibition leading to increased mortality via reduced posterior swim bladder inflation* | Embryo (High) | *Pimephales promales* (High), *Danio rerio* (High) | Unspecific (Moderate) | EAGMST Approved |
| 158 | *Deiodinase 1 inhibition leading to increased mortality via reduced anterior swim bladder inflation* | Larvae (High) | *Pimephales promales* (High), *Danio rerio* (High) | Unspecific (Moderate) | EAGMST Approved |
| 159 | *Thyroperoxidase inhibition leading to increased mortality via reduced anterior swim bladder inflation* | Larvae (High) | *Pimephales promales* (High), *Danio rerio* (High) | Unspecific (Moderate) | EAGMST Approved |
| 162 | *Enhanced hepatic clearance of thyroid hormones leading to thyroid follicular cell adenomas and carcinomas in the rat and mouse* | N.A. | *Rattus sp.* (High), *Mus musculus* (Moderate) | N.A. | N.A. |
| 165 | *Antiestrogen activity leading to ovarian adenomas and granular cell tumors in the mouse* | N.A. | *Mus musculus* (Moderate), *Rattus norvegicus* (High) | Female (High) | N.A. |
| 167 | *Early-life estrogen receptor activity leading to endometrial carcinoma in the mouse.* | Fetal to Parturition (Moderate), Juvenile (Moderate) | *Mus musculus* (High) | Female (High) | N.A. |
| 175 | *Thyroperoxidase inhibition leading to altered amphibian metamorphosis* | Development (high) | *Xenopus laevis* (high) | Unspecific (moderate) | N.A. |
| 176 | *Sodium Iodide Symporter (NIS) Inhibition leading to altered amphibian metamorphosis* | Development (high) | *Xenopus laevis* (high) | Unspecific (high) | N.A. |
| 188 | *Iodotyrosine deiodinase (IYD) inhibition leading to altered amphibian metamorphosis* | Development (high) | *Xenopus laevis* (low) | Unspecific (high) | N.A. |
| 189 | *Type I iodothyronine deiodinase (DIO1) inhibition leading to altered amphibian metamorphosis* | Development (high) | *Xenopus laevis* (low) | Unspecific (high) | N.A. |
| 190 | *Type II iodothyronine deiodinase (DIO2) inhibition leading to altered amphibian metamorphosis* | Development (high) | Xenopus laevis (moderate) | Unspecific (high) | N.A. |
| 191 | *Type III iodotyrosine deiodinase (DIO3) inhibition leading to altered amphibian metamorphosis* | Development (high) | *Xenopus laevis* (high) | Unspecific (high) | N.A. |
| 200 | *Estrogen receptor activation leading to breast cancer* | Not Otherwise Specified (High) | *Homo sapiens* (High), *Felis catus* (High), *Canis lupus familiaris* (High) | Unspecific (high) | N.A. |
| 271 | *Inhibition of thyroid peroxidase leading to impaired fertility in fish* | Adult, reproductively mature (High) | Fish (High) | Female (High) | Under Development |
| 288 | *Inhibition of 17α-hydrolase/C 10,20-lyase (Cyp17A1) activity leads to birth reproductive defects (cryptorchidism) in male (mammals)* | Development (high) | *Homo sapiens*, *Rattus norvegicus* (moderate) | Male (high) | N.A. |
| 289 | *Inhibition of 5α-reductase leading to impaired fecundity in female fish* | 3 to < 6 months (Moderate) | Fish (Moderate) | Female (Moderate) | Under Development |
| 295 | *Early-life stromal estrogen receptor activation by endocrine disrupting chemicals in the mammary gland leading to enhanced cancer risk* | N.A. | N.A. | N.A. | Under Development |
| 300 | *Thyroid Receptor Antagonism and Subsequent Adverse Neurodevelopmental Outcomes in Mammals* | During brain development (High) | *Homo sapiens* (High), *Mus musculus* (High) | Male (High), Female (High) | Under Development |
| 305 | *5α-reductase inhibition leading to short anogenital distance (AGD) in male (mammalian) offspring* | Pregnancy (high) | *Rattus norvegicus* (high); *Homo sapiens*, *Mus musculus* (moderate) | Male (high) | Under development |
| 306 | *Androgen receptor (AR) antagonism leading to short anogenital distance (AGD) in male (mammalian) offspring* | Pregnancy (high) | *Rattus norvegicus* (high); *Homo sapiens*, *Mus musculus* (moderate) | Male (high) | Under development |
| 307 | *Decreased testosterone synthesis leading to short anogenital distance (AGD) in male (mammalian) offspring* | Foetal, pregnancy (high) | *Rattus norvegicus* (high); *Homo sapiens*, *Mus musculus* (moderate) | Male (high) | Under development |
| 309 | *Luteinizing hormone receptor antagonism leading to reproductive dysfunction* | 3 to < 6 months (Not specified) | Fish (Moderate) | Female (High) | N.A. |
| 314 | *Binding to estrogen receptor (ER)-α in immune cells leading to exacerbation of systemic lupus erythematosus (SLE)* | All life stages (Moderate) | *Homo sapiens* (Moderate) | Mixed (High) | Under Development |
| 321 | *Reduced environmental pH leading to thinner shells in Mytilus edulis* | N.A. | N.A. | N.A. | N.A. |
| 344 | *Androgen receptor (AR) antagonism leading to nipple retention (NR) in male (mammalian) offspring* | N.A. | N.A. | N.A. | N.A. |
| 345 | *Androgen receptor (AR) antagonism leading to decreased fertility in females* | N.A. | N.A. | N.A. | N.A. |
| 346 | *Aromatase inhibition leads to male-biased sex ratio via impacts on gonad differentiation* | Development (High) | *Danio rerio* (High), *Oreochromis niloticus* (High), *Oncorhynchus tshawytscha* (Low), *Pimephales promelas* (Low), *Dicentrarchus labrax* (Low) | Unspecific (High) | N.A. |
| 348 | *Inhibition of 11β-Hydroxysteroid Dehydrogenase leading to decreased population trajectory* | Adult, reproductively mature (Moderate) | Fish (High) | Male (High) | Under Development |
| 349 | *Inhibition of 11β-hydroxylase leading to ecreased population trajectory* | Adult, reproductively mature (High) | Fish (Moderate) | Mixed (High) | Under Development |
| 366 | *Competitive binding to thyroid hormone carrier protein transthyretin (TTR) leading to altered amphibian metamorphosis* | N.A. | N.A. | N.A. | N.A. |
| 367 | *Competitive binding to thyroid hormone carrier protein thyroid binding globulin (TBG) leading to altered amphibian metamorphosis* | N.A. | N.A. | N.A. | N.A. |
| 372 | *Androgen receptor antagonism leading to testicular cancer* | N.A. | N.A. | N.A. | N.A. |
| 376 | *Androgen receptor agonism leading to male-biased sex ratio* | Developmental (High) | *Danio rerio* (High), *Oryzias latipes* (Low), *Pimephales promelas* (Low), *Ictalurus punctatus* (Low), *Oreochromis niloticus* (Low), *Oncorhynchus tshawytscha* (Low) | Unspecific (High) | N.A. |
| 393 | *AOP for thyroid disorder caused by triphenyl phosphate* | N.A. | N.A. | N.A. | N.A. |
| 401 | *G protein-coupled estrogen receptor 1 (GPER) signal pathway in the endocrine disrupting effect* | N.A. | N.A. | N.A. | N.A. |
| 402 | *Thyroid peroxidase (TPO) inhibition leads to periventricular heterotopia formation in the developing rat brain* | N.A. | N.A. | Mixed (High) | N.A. |
| 440 | *Hypothalamus estrogen receptors activity suppression leading to ovarian cancer via ovarian epithelial cell hyperplasia* | Adult, reproductively mature (High) | *Homo sapiens* (High)*, Rattus norvegicus* (High), *Mus sp.* (High) | Female (High) | Under Development |
| 443 | *Alcohol Induced DNA damage and mutations leading to Metastatic Breast Cancer* | Adult, reproductively mature (Moderate) | Human and other cells in culture (High)*, Homo sapiens* (Moderate)*, Mus sp.* (Moderate)*, Rattus norvegicus (*Moderate)*, Dirofilaria immitis* (Moderate)*, Saccharomyces cerevisiae* (Moderate)*,* | Female (High) | Under Development |
| 445 | *Estrogen Receptor Alpha Agonism leads to Impaired Reproduction* | N.A. | N.A. | N.A. | N.A. |
| 465 | *Alcohol dehydrogenase leading to reproductive dysfunction* | N.A. | N.A. | N.A. | N.A. |
| 476 | *Adverse Outcome Pathways diagram related to PBDEs associated male reproductive toxicity* | N.A. | N.A. | N.A. | N.A. |
| 477 | *Androgen receptor (AR) antagonism leading to hypospadias in male offspring* | N.A. | N.A. | N.A. | N.A. |

1. [↑](#footnote-ref-1)
2. 1 The information for AOP IDs 7-376 was collected on 2022-09-13 and AOP titles may have been modified since the initial search for data in the AOP-Wiki on 2021-07-04. AOP IDs higher than 376 were collected on 2023-02-21, and may also have been changed since. [↑](#endnote-ref-1)
