# Supplementary figures and images for: Development of a data-driven approach to Adverse Outcome Pathway network generation: a case study on the EATS-modalities
Source: Front Toxicol. 2023 May 9;5:1183824. doi: 10.3389/ftox.2023.1183824 (PMC10203404; doi:10.3389/ftox.2023.1183824)

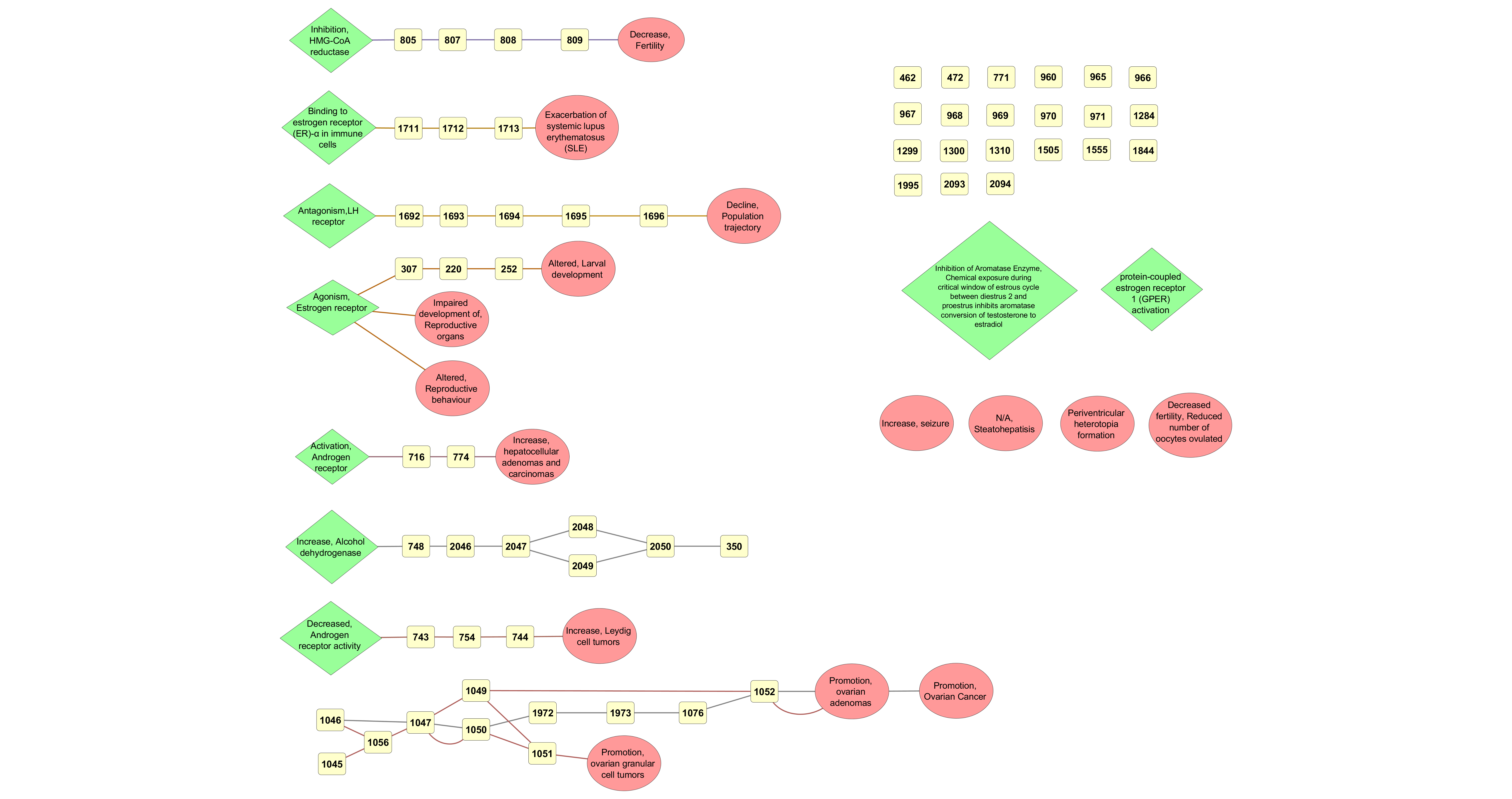

Supplement: Supplementary file 3 [file Image2.PNG]

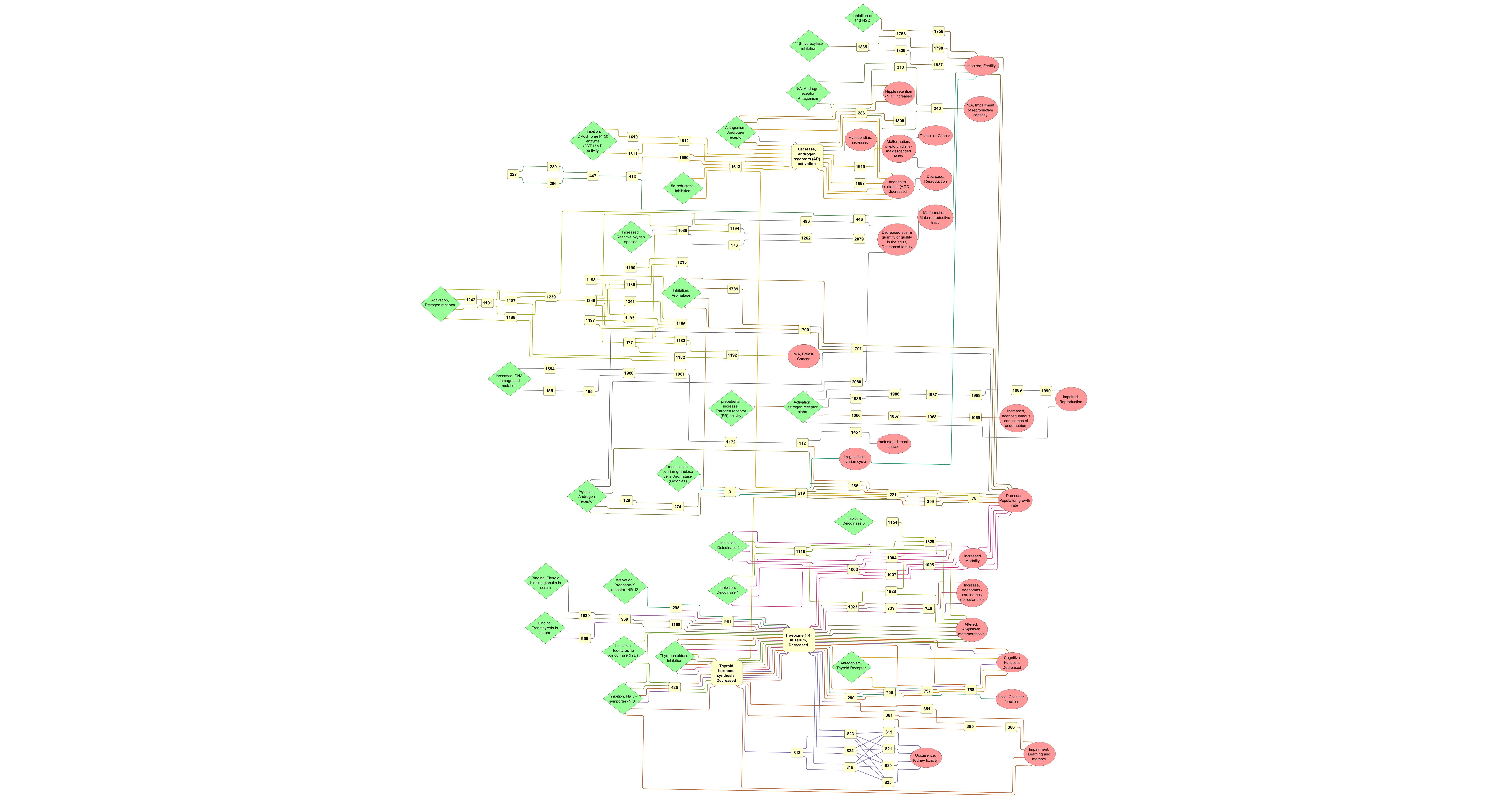

Supplement: Supplementary file 5 [file Image1.PNG]
